# Exercise Shifts Hypothetical Food Choices toward Greater Amounts and More Immediate Consumption

**DOI:** 10.3390/nu13020347

**Published:** 2021-01-24

**Authors:** Karsten Koehler, Safiya E. Beckford, Elise Thayer, Alexandra R. Martin, Julie B. Boron, Jeffrey R. Stevens

**Affiliations:** 1 Department of Sport and Health Sciences, Technical University of Munich, 80992 Munich, Germany; alexandra.martin@tum.de; 2 Department of Nutrition and Health Sciences, University of Nebraska-Lincoln, Lincoln, NE 68583, USA; safiyabeckford@gmail.com; 3 Department of Psychology, University of Nebraska-Lincoln, Lincoln, NE 68588, USA; eliserthayer@gmail.com (E.T.); jeffrey.r.stevens@gmail.com (J.R.S.); 4 Department of Gerontology, University of Nebraska Omaha, Omaha, NE 68182, USA; jboron@unomaha.edu

**Keywords:** aerobic exercise, food choice, compensatory eating, food amount

## Abstract

Although exercise modulates appetite regulation and food intake, it remains poorly understood how exercise impacts decision-making about food. The purpose of the present study was to assess the impact of an acute exercise bout on hypothetical choices related to the amount and timing of food intake. Forty-one healthy participants (22.0 ± 2.6 years; 23.7 ± 2.5 kg/m^2^, 56% female) completed 45 min of aerobic exercise and a resting control condition in randomized order. Food amount preferences and intertemporal food preferences (preference for immediate vs. delayed consumption) were assessed using electronic questionnaires with visual food cues. Compared to rest, exercise resulted in a greater increase in the food amount selected, both immediately post-exercise (+25.8 ± 11.0 vs. +7.8 ± 11.0 kcal/item, *p* = 0.02) and 30 min post-exercise (+47.3 ± 12.4 vs. +21.3 ± 12.4 kcal/item, *p* = 0.005). Exercise further resulted in a greater increase in the preference for immediate consumption immediately post-exercise (+0.23 ± 0.10 vs. +0.06 ± 0.10; *p* = 0.03) and 30 min post-exercise (+0.30 ± 0.12 vs. +0.08 ± 0.12; *p* = 0.01). Our findings demonstrate that a single bout of aerobic exercise shifts hypothetical food choices toward greater amounts and more immediate consumption, highlighting the importance of the timing of food choices made in the exercise context.

## 1. Introduction

Regular exercise and a balanced diet are both key staples of a healthy lifestyle. The beneficial effects of exercise on many physiological and psychological conditions are well-established [1,2,3], but its impact on food intake remains ambiguous. On the one hand, there is ample evidence that individuals who exercise regularly consume a healthier diet [4,5] and that exercise improves appetite regulation and control over energy intake [6], modifies the sensitivity to sensory cues [7], and alters the reward value of food [8], thereby protecting from overeating and weight gain [9]. On the other hand, the palatability of energy-dense foods has been shown to be increased in the immediate post-exercise state [10], individuals are less likely to choose a heathier snack option following exercise when compared to prior to their workout [11], and very high levels of exercise and physical activity are linked with a greater consumption of unhealthy food items such as added sugar [12]. The increase in food intake following exercise or physical activity, a phenomenon often termed compensatory eating [13], has been reported to occur in up to 75% of exercisers [14] and is understood as a primary barrier for weight loss [15]. While the mechanisms for compensatory eating remain not fully understood, it most likely serves to protect from the loss of lean mass [6,16].

Experiments on the impact of exercise on food intake typically involve the assessment of ad libitum food intake after exposing individuals to defined periods of exercise or rest [17]. While ad libitum food intake is an important outcome, this approach is limited by the fact that ad libitum food intake directly alters energy balance, thereby interfering with subsequent food intake regulation. As a result, ad libitum food intake can be measured only once, typically at the study endpoint.

Considerably less is known about the time course of food intake preferences, although changes in subjective perceptions of hunger [18], appetite-regulating peptides [19], and palatability [10] suggest that food intake regulation is not static and is subject to change over the course of an exercise bout. The aspect of time is particularly relevant in the context of intertemporal choices, which refer to decisions between options that result in outcomes realized at different times [20] and often involve tradeoffs between immediate rewards (i.e., consumption of palatable, energy-dense food) and long-term benefits (i.e., improved health). In general, temptations for immediate gratification are difficult to control [21], and a greater tendency for immediate gratification has been linked to unhealthy eating patterns and obesity [22,23]. Considering the established effects of exercise on food intake regulation and its ability to induce compensatory eating [24], it is likely that exercise shifts the intertemporal preference toward more immediate gratification. To our knowledge, intertemporal food choices have not been studied previously in the context of exercise, although their understanding is critical in efforts to construct strategies to “nudge” people into making healthier long-term food choices [21].

To address this gap in the literature, the goal of the present study was to determine the impact of exercise on food choices with particular reference to the time course of changes in food amount preferences and intertemporal choices. To achieve this objective, we conducted a randomized crossover exercise trial using a food choice paradigm consisting of a series of hypothetical choices. Considering that hypothetical food choices can be as valid as actual choices [25], this approach offers several advantages, including the ability to measure food intake preferences at various time points over the course of an experiment and to quantify shifts in preferences for food amount, type, and intertemporal choices on separate scales. In agreement with previous literature, we hypothesized exercise to shift food choices toward greater amounts and more immediate consumption. Specifically, we predicted that increases in food amount preference and the preference for immediate consumption over the course of an exercise bout would exceed changes over the same timeframe at rest and that these changes would persist beyond the immediate post-exercise state.

## 2. Materials and Methods

### 2.1. Study Design

In a randomized, two-way crossover study, participants completed two separate study visits. They were randomly assigned to either a 45 min exercise bout or an equally long period of rest for their first visit and completed the other study condition in their second visit. All participants gave their written informed consent for inclusion before they participated in the study. The study was conducted in accordance with the Declaration of Helsinki, and the protocol was approved by the Institutional Review Board of the University of Nebraska-Lincoln (project number 17239).

### 2.2. Participants

Volunteers for this study were recruited from three University of Nebraska campuses and their surrounding communities via fliers and word-of-mouth. Inclusion of interested individuals was assessed in a two-step process, including the completion of an online survey followed by an in-depth screening to determine final eligibility. Participants were included if they were 19–29 years of age, had a body mass index not indicative of underweight (<18.5 kg/m^2^) or obesity (>30 kg/m^2^), exercised regularly (≥1 bout/week), and were weight-stable within the past 6 months (±2.5 kg). Exclusion criteria included pregnancy, smoking, any medical condition or use of medication that could affect appetite or present any contraindications to exercise, a history of or current eating disorder, or a self-reported inability to exercise at a moderate intensity for 45 min. In addition, participants who were allergic to or strongly disliked any of the food cues used in this study were excluded prior to participation.

### 2.3. Preliminary Assessments

Participants completed two preliminary visits, which involved the assessment of anthropometric data, body composition, diet and exercise habits, and health history, as well as completion of an exercise test. Participants were weighed to the nearest 0.1 kg and height was taken to the nearest 0.1 cm using a digital scale and stadiometer (Seca, Hamburg, Germany) with a standard outfit of t-shirt and gym shorts. Peak oxygen uptake (VO_2peak_) was assessed using an incremental exercise test on a bicycle ergometer (LC6, Monark, Vansbro, Sweden). Participants began cycling at a resistance of 60 W for 3 min, and the work rate was increased by 35 W every 3 min until exhaustion [26]. Exhaustion was operationally defined when at least two of the following were met: (1) Heart rate of ≥90% of age-predicted maximal heart rate, (2) a respiratory exchange ratio ≥1.1, (3) a rate of perceived exertion ≥19, (4) a plateau in oxygen uptake despite increasing workload. Throughout the test, gas exchange was measured with a metabolic cart (Quark CPET, COSMED, Rome, Italy) and heart rate was monitored through telemetry (Polar, Kempele, Finland).

### 2.4. Study Conditions

Prior to each study condition, participants arrived at the lab between 06:30 and 10:00 following an overnight fast and alcohol and caffeine abstention for at least 24 h. Participants were further asked to abstain from exercise and strenuous physical activity the day before and the morning of their visits, with compliance monitored via accelerometry (GT3X+, Actigraph, Pensacola, FL, USA). During their first study condition visit, participants completed a 24 h diet recall using an Automated Self-Administered 24-h Dietary Assessment Tool (ASA24, National Cancer Institute, Bethesda, MD, USA). Following this first visit, participants were given a copy of their recall and were instructed to replicate the diet as closely as possible the day prior to their second visit.

Upon arrival at the lab, participants were provided with a small, standardized snack (commercially available cereal bar; 240 kcal, 8 g of protein) and 8 ounces of bottled water so that participants did not exercise in a completely fasted state while avoiding a longer wait period prior to the onset of exercise to digest a larger meal, thereby mimicking previous laboratory experiments [17]. After resting for 30 min in a seated position, participants completed surveys about their subjective ratings of hunger and fullness, preferred food amount for consumption, and choices between foods varying in the time of consumption. Participants then exercised on a bicycle ergometer (LC6, Monark, Vansbro, Sweden) for 45 min at an intensity equivalent to 60% of their VO_2peak_, an intensity that has previously been found to increase ad libitum food intake [19,27]. Trained lab personnel monitored participants’ heart rate and ratings of perceived exertion [28] at regular intervals throughout the exercise. Immediately following the exercise, participants completed the surveys for a second time, rested for 30 min, and completed the surveys for a third time. The resting condition was identical to the exercise condition, except that the 45 min exercise bout was substituted for a rest period, during which participants sat quietly in a chair for 45 min. Participants were allowed to listen to music or watch pre-approved TV programs that did not contain any images of or references to food.

### 2.5. Surveys

Participants completed all surveys in electronic format on a handheld tablet (iPad, Apple, Cupertino, CA, USA) at three time points during each condition, prior to the exercise bout/rest period (“pre”), immediately upon completion of the exercise bout/rest period (“post”), and after an additional 30 min of recovery/rest (“post + 30”). At each time point, participants first rated their subjective perception of hunger, fullness, thirst, nausea, and stress on a condensed visual analog scale from 0 to 10. Participants then reported their food amount preference and temporal food preference by responding to a series of hypothetical questions involving visual food cues. To incentivize the participants to make realistic food choices, participants were informed that they would receive one of the selected foods as a reward immediately after completing the study condition. This information was provided only at the beginning of the survey and was not directly related to a specific choice. All participants received the same food item (i.e., pizza) in excess of the largest option for the amount preference at the end of each condition. The food items in the survey included eight food items with varying palatability and energy density (sweet, nonsweet, high fat, low fat). The food selection was guided by the Leeds Food Preference Questionnaire [29,30], which was adjusted to a North American population under consideration of palatability and macronutrient and energy content.

**Figure 1:**
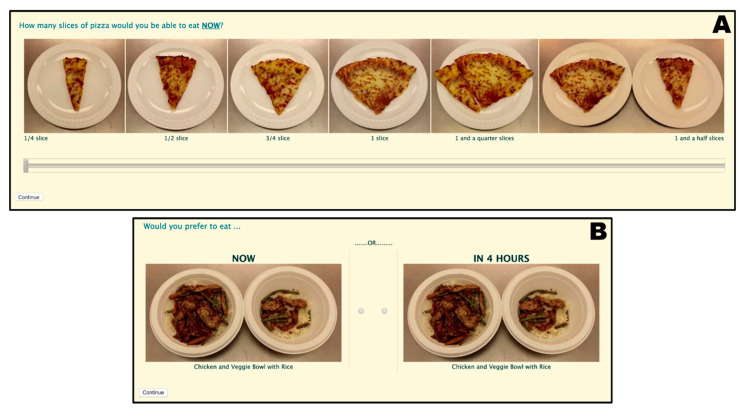
Examples of questions employed for the quantification of food amount preferences (**A**) and intertemporal food preferences (**B**).

Participants reported their food amount preferences by choosing their preferred portion size of each food item (Figure 1A). The food cues represented common portions ranging from 75 to 450 kcal and included pictures of each portion, as well as portion descriptions (e.g., ½ slice of pizza); no caloric information was provided to the participants. Food amount preferences were assessed both for immediate and delayed consumption. For delayed consumption, which was defined as consumption in 4 h, survey materials informed participants that they should make their decision under the assumption that they would not be able to eat anything until then.

Intertemporal food preferences were assessed by asking participants to choose between two food options available for either immediate or delayed consumption (Figure 1B). Again, delayed consumption was defined as consumption in 4 h without any food until then. Temporal food preferences were collected for all possible combinations of food items available in the previous food amount preference assessment but with a standardized food amount such that each portion represented 225 kcal.

### 2.6. Data Processing and Statistical Analysis

All data were analyzed using R statistical software v3.6.0 (R Core Team, Vienna, Austria) [31] and R-packages (see Data Availability Statement). Unless otherwise stated, data are reported as means with 95% within-subject confidence intervals. Changes in food amount preferences were calculated by subtracting preferred amounts at the beginning of each condition (pre) from preferred amounts after completion of the exercise/rest period (post) and 30 min after completion of the exercise/rest period (post + 30). As a result, positive values indicate an increase and negative values a decrease in preferred amount over time. Changes in food amount preference were calculated both for immediate and delayed consumption. For intertemporal food preferences, the proportion of choices for immediate consumption was calculated. Changes in choice proportions were calculated by subtracting proportions at the beginning of each condition (pre) from proportions both after completion of the exercise/rest period (post) and 30 min after completion of the exercise/rest period (post + 30). Consequently, positive values indicate an increased preference for immediate consumption, whereas negative values indicate an increased preference for delayed consumption over time.

Differences in subjective perception of hunger, fullness, thirst, and stress between conditions and over time were analyzed using repeated-measures analysis of variance (ANOVA). As ANOVAs for fullness and hunger violated assumptions of sphericity, results are reported with Greenhouse–Geisser- and Huynh–Feldt-corrected *p*-values. The ANOVA for nausea resulted in severe violations of model assumptions and is thus not reported. Repeated-measures ANOVAs were also used to assess changes in food amount preference and intertemporal food preference across conditions (exercise vs. rest) and delays (now vs. later). As ANOVAs including food type (low fat/nonsweet, low fat/sweet, high fat/nonsweet, and high fat/sweet) violated the model assumption for normally distributed residuals and did not contribute to any main effects or interactions (*p* > 0.12), food type was excluded from all subsequent analyses and data were analyzed and displayed in aggregate format across all food types. Food amount preferences and intertemporal food preferences for each food type are presented in Figures S1–S4.

## 3. Results

### 3.1. Participant Characteristicss

Data were collected from 48 participants between October 2017 and December 2018. Data from seven participants were excluded retrospectively because participants inadvertently exercised at a greater intensity or failed to meet updated age qualifications (see CONSORT diagram, Supplementary Digital Content S1). We analyzed data from the remaining 41 participants (23 women, 18 men). On average, these participants were 22.0 ± 2.6 years old, had a body-mass index of 23.7 ± 2.5 kg/m^2^, and had a VO_2peak_ of 37.3 ± 6.2 mL/kg/min.

### 3.2. Subjective Perceptions

**Figure 2:**
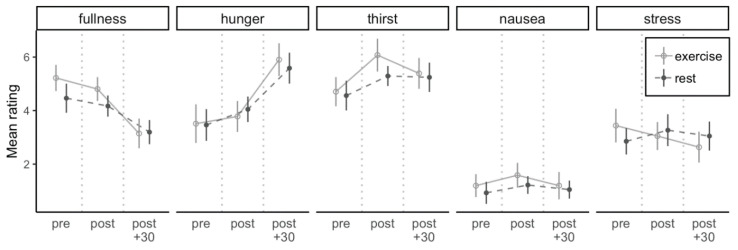
Subjective ratings of fullness, hunger, thirst, nausea, and stress at each time point (pre, post, post + 30) for both conditions. Circles represent means and error bars represent 95% within-subject confidence intervals.

Figure 2 illustrates the time course of ratings of fullness, hunger, thirst, nausea, and stress. There were main effects of time for fullness, hunger, and thirst (all *p* < 0.001, $\eta_{G}^{2}$ ≥ 0.04) but not on stress (*p* = 0.29, $\eta_{G}^{2}$ = 0.004), with hunger and thirst increasing and fullness decreasing over time. There was no main condition effect for fullness, hunger, thirst, and stress (all *p* ≥ 0.08, $\eta_{G}^{2}$ ≤ 0.01). There was no interaction of condition and time for fullness, hunger, and thirst (all *p* ≥ 0.08, $\eta_{G}^{2}$ ≤ 0.006), but there was an interaction of condition and time on stress (*p* = 0.02, $\eta_{G}^{2}$ = 0.008), which decreased within the exercise conditions from pre to post + 30 (3.4 ± 0.6 vs. 2.6 ± 0.6; *p* = 0.04).

### 3.3. Food Amount Preference

**Figure 3:**
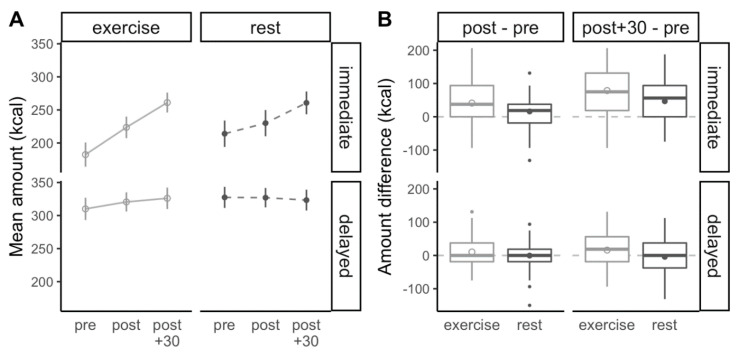
(**A**) Food amount preferences for immediate and delayed consumption at each time point during the experiment. (**B**) Changes in food amount preference from before to immediately after the exercise/rest bout (post–pre) and from before to 30 min after completion of the exercise/rest bout (post + 30–pre) for immediate and delayed consumption for condition interactions (exercise vs. rest). Positive values indicate an increase in the food amount preferences after the exercise or rest condition, and negative values represent a reduction in food amount preferences. Circles represent means and error bars represent 95% within-subject confidence intervals, horizontal bars represent medians, boxes represent interquartile ranges, whiskers represent observations within 1.5 times the interquartile range, and outer points represent outliers.

Figure 3 illustrates the changes in food amount preferences for immediate and delayed consumption over the course of both conditions. There were main effects of condition (exercise vs. rest) for changes from pre to post (*p* = 0.02, $\eta_{G}^{2}$ = 0.03) and for changes from pre to post + 30 (*p* = 0.005, $\eta_{G}^{2}$ = 0.05), indicating that the amount selected for consumption was increased immediately after exercise (+25.8 ± 11.0 vs. +7.8 ± 11.0 kcal per item) and after resting for 30 min post-exercise (+47.3 ± 12.4 vs. +21.3 ± 12.4 kcal per item). There was also a main effect of delay both for changes from pre to post (*p* < 0.001, $\eta_{G}^{2}$ = 0.04) and from pre to post + 30 (*p* < 0.001, $\eta_{G}^{2}$ = 0.19), with a greater increase in the amount selected for immediate vs. delayed consumption immediately after exercise (+28.6 ± 9.1 vs. +5.0 ± 9.1 kcal per item) and after resting for 30 min post-exercise (+62.7 ± 10.8 vs. +5.9 ± 10.8 kcal per item). There was no interaction of condition and delay for changes from pre to post (*p* = 0.22, $\eta_{G}^{2}$ = 0.004) and from pre to post + 30 (*p* = 0.31, $\eta_{G}^{2}$ = 0.002).

### 3.4. Intertemporal Food Preference

**Figure 4:**
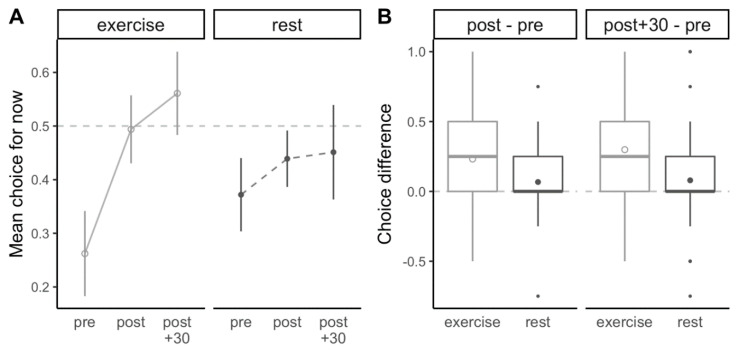
(**A**) Intertemporal food preference at each time point during the experiment. Values >0.5 indicate a greater preference for immediate consumption, and values <0.5 indicate a greater preference for delayed consumption. (**B**) Changes in intertemporal food preferences from before to immediately after the exercise/rest bout (post-pre) and from before to 30 min after completion of the exercise/rest bout (post + 30–pre). Positive values indicate an increase in the preference for immediate consumption, and negative values indicate an increase in the preference for delayed consumption. Circles represent means and error bars represent 95% within-subject confidence intervals, horizontal bars represent medians, boxes represent interquartile ranges, whiskers represent observations within 1.5 times the interquartile range, and outer points represent outliers.

Figure 4 illustrates the changes in intertemporal food preferences over the course of both conditions. When compared to rest, the increase in the proportional preference for immediate vs. delayed consumption was significantly greater immediately after exercise (+0.23 ± 0.10 vs. +0.06 ± 0.10; *p* = 0.03, d = 0.36), as well as 30 min post-exercise (+0.30 ± 0.12 vs. +0.08 ± 0.12; *p* = 0.01, d = 0.40).

## 4. Discussion

The overall goal of this study was to determine the impact of an acute exercise bout on food amount preference and preference for immediate consumption. Using a novel paradigm consisting of a series of hypothetical food choices, results from this randomized crossover trial demonstrate that food choices shift over the course of a 45 min exercise bout toward greater amounts and more immediate consumption when compared to rest and that these changes persisted for at least 30 min post-exercise.

Our first observation, according to which 45 min of cycling at a moderate intensity increased the amount of food selected for consumption, is in agreement with a large body of research on compensatory eating following exercise. When presented with a series of questions prompting them to select the amount of food they would be able to consume, participants in the present study increased the amount selected by on average 25.8 kcal per food item over the course of the 45 min exercise, which was significantly greater than the 7.8 kcal-increase per item that occurred during the control condition. Thirty minutes post-exercise, the increase in the amount selected for consumption grew to 47.3 kcal per food item, while the increase at the same time point in the control condition was only 21.3 kcal per food item. Albeit differences of 18–26 kcal per item may appear small, the gap between exercise and rest amounts to 7% (immediately post-exercise) to 10% (30 min post-exercise) when expressed relative to the amount chosen before exercise. It should further be noted that these increases represent the average increase occurring over multiple hypothetical choices between the same food varying in portion size and caloric content, ranging from very small (~75 kcal/item) to moderate size (~450 kcal/item), thereby providing a robust measure of food amount preference across foods with varying properties. Even though this approach differs substantially from the method typically used to quantify compensatory eating (i.e., ad libitum intake during a test meal), differences between conditions were within the same order of magnitude as the 48 kcal-gap in energy intake reported in a meta-analysis of 51 controlled experiments comparing exercise and rest [17]. To our knowledge, the only other study employing a similar approach of integrating multiple hypothetical choices reported a reduction in food amount selected after 1 h of low-intensity exercise (walking) when compared to rest, although this effect disappeared within 60 min after exercise completion. Despite these differences, visual inspection of the data from Farah et al. suggests a reduction in food amount preference prior to exercising [18], which is in agreement with our observations.

While there was no significant interaction between amount and delay, the increase in food amount preference was much more pronounced when participants were asked to choose food for immediate consumption, as shown by increases of +29 kcal per item (immediately post-exercise) and +63 kcal per item (30 min post-exercise) for immediate consumption compared to increases between 5 and 6 kcal per item for delayed consumption. This observation is in agreement with our results related to intertemporal food choices, which demonstrate a marked increase in the preference for immediate food consumption in the post-exercise state. When prompted to select foods either for immediate or delayed consumption, participants were on average 23% (immediately post-exercise) to 30% (30 min post-exercise) more likely to select a food item for immediate consumption. Similarly to food amount preference, this shift resulted from the combination of a reduced preference for immediate consumption prior to exercising and an increased preference for immediate consumption after exercise completion when compared to the same time points during the rest condition, and was more pronounced 30 min post-exercise. To our knowledge, we are the first to report such a shift in intertemporal food choices in the context of exercise. Previous authors have predominantly used monetary paradigms to quantify the impact of exercise on intertemporal choices [32,33], although relationships between obesity-related variables and intertemporal choices have been established for both money and food [34]. With our focus on food choices and our intention to mimic real-life choices as closely as possible, we elected to frame our questions related to intertemporal food choices in a time frame that resembles human consumption patterns, i.e., between immediate consumption and consumption for the next meal.

Another important finding from the present study is that the impact of exercise on food amount preference and preference for immediate consumption extends beyond the immediate post-exercise state and was actually more pronounced 30 min after completion of the exercise bout. This observation may at least be partly a result of a transient suppression of appetite during and immediately after exercise [35]. Although we failed to detect significant changes in subjective ratings of hunger or fullness immediately following the exercise bout, our results suggest that—even if it exists—this phenomenon is only short-lived and does not meaningfully reduce food amount preference and intertemporal preference. This observation is further supported by previous reports of self-reported ratings of appetite and hunger returning to or exceeding resting levels within 30 min post-exercise [19].

The present trial served as a first proof-of-principle study for the use of our food choice paradigm to measure transient shifts in decision-making about food in the context of exercise. Although the benefits of using a series of hypothetical food choices include the ability to repeatedly collect data without interfering with the subjects’ metabolic state and the ability to quantify shifts in preferences for food amount, type, and intertemporal choices on separate scales, our approach is not without limitations. The most obvious limitation is the use of hypothetical rather than real food choices, although previous research supports that hypothetical food choices can be equally valid [25] and have been used to determine shifts in prospective food consumption by others [18]. Nevertheless, future work should ensure that our results generalize to actual choices. The use of a series of hypothetical food choices further provides no direct measure of ad libitum intake, which can be used to quantify relative energy intake. This outcome, which relates ad libitum intake during a test meal to the energy expended while exercising, is frequently reported in exercise studies and shows large effect sizes when compared to rest [17]. However, the aforementioned increases in preference and the energy expended during the 45 min exercise bout, which was on average 343 ± 90 kcal, were within the same order of magnitude as previously reported [17]. While we acknowledge the inability to quantify relative energy intake as a limitation, we believe that the ability to collect data at multiple time points allows us to pinpoint how ad libitum intake—and consequently relative energy intake—could be shifted even further, for example, by making individuals choose their next meal prior to exercising. Further, as our food amount preference data represent the average of multiple choices between real-life options of the same foods varying in portion size and caloric content, it potentially provides more robust evidence of post-exercise shifts in food preferences. While the delivery of hypothetical food choices in the context of exercise is relatively novel in itself, our approach builds on prior knowledge. The food items selected for the food choice survey mirrored items previously used in the Leeds Food Preference Questionnaire [29,30], which we adapted to a North American palate. Although our survey encompassed foods with different rewarding food properties, such as sweet or fatty, we did not include food type into our final analysis, due to model violations and reporting aggregate data across all food types in order to focus on our primary outcomes related to amount and intertemporal preferences.

Given the exploratory nature, the present study was conducted in a sample of young, healthy adults who were nonobese and of average physical fitness, a group in whom food choices have been shown to vary between the pre- and post-exercise state before [11]. Future experiments are required in other populations, such as individuals seeking weight loss through exercise for whom compensatory weight loss is considered a major barrier [15]. The exercise characteristics, such as mode (cycling), intensity (moderate), and duration (45 min), used in the present experiment were determined based on previous studies reporting shifts in ad libitum food intake in the post-exercise state [19,27], as well as pragmatic reasons such as a lower susceptibility to familiarization and training effects when compared to other exercise types such as running or resistance training. Nevertheless, the food choice responses to other exercise modes and modalities need to be established systematically [17].

## 5. Conclusions

Our present findings suggest that compensatory increases in food intake following exercise are the result of an increased food amount preference coupled with an increased preference for more immediate food consumption. The fact that shifts in food choices occur over the course and in the aftermath of an exercise bout highlights the importance of the timing of food choices in the context of exercise. Building on our findings, which suggest that food choices, when made prior to exercising, may be less vulnerable to compensatory mechanisms, further research is needed to determine whether this strategy can improve the weight loss success of exercise interventions. Considering that weight loss is a key motive for exercise participation in the first place and failure to achieve the desired weight loss predicts drop-outs [36], our findings may also improve long-term adherence to exercise programs and thereby contribute to favorable health outcomes beyond weight loss.

## Data Availability

Data and R scripts are available at https://osf.io/tqcwx/.

## Supplementary Materials

The following are available online at https://www.mdpi.com/2072-6643/13/2/347/s1, Supplementary Digital Content S1: CONSORT diagram, Figure S1: Mean amount preferences for immediate and delayed consumption by food type, Figure S2: Changes in food amount preference by food type, Figure S3: Intertemporal food preference by food type, Figure S4. Changes in intertemporal food preferences by food type. A preprint of the paper was published online at https://doi.org/10.31234/osf.io/3z2er.
