# Supplementary figures and images for: Exercise Shifts Hypothetical Food Choices toward Greater Amounts and More Immediate Consumption
Source: Nutrients. 2021 Jan 24;13(2):347. doi: 10.3390/nu13020347 (PMC7911174; doi:10.3390/nu13020347)

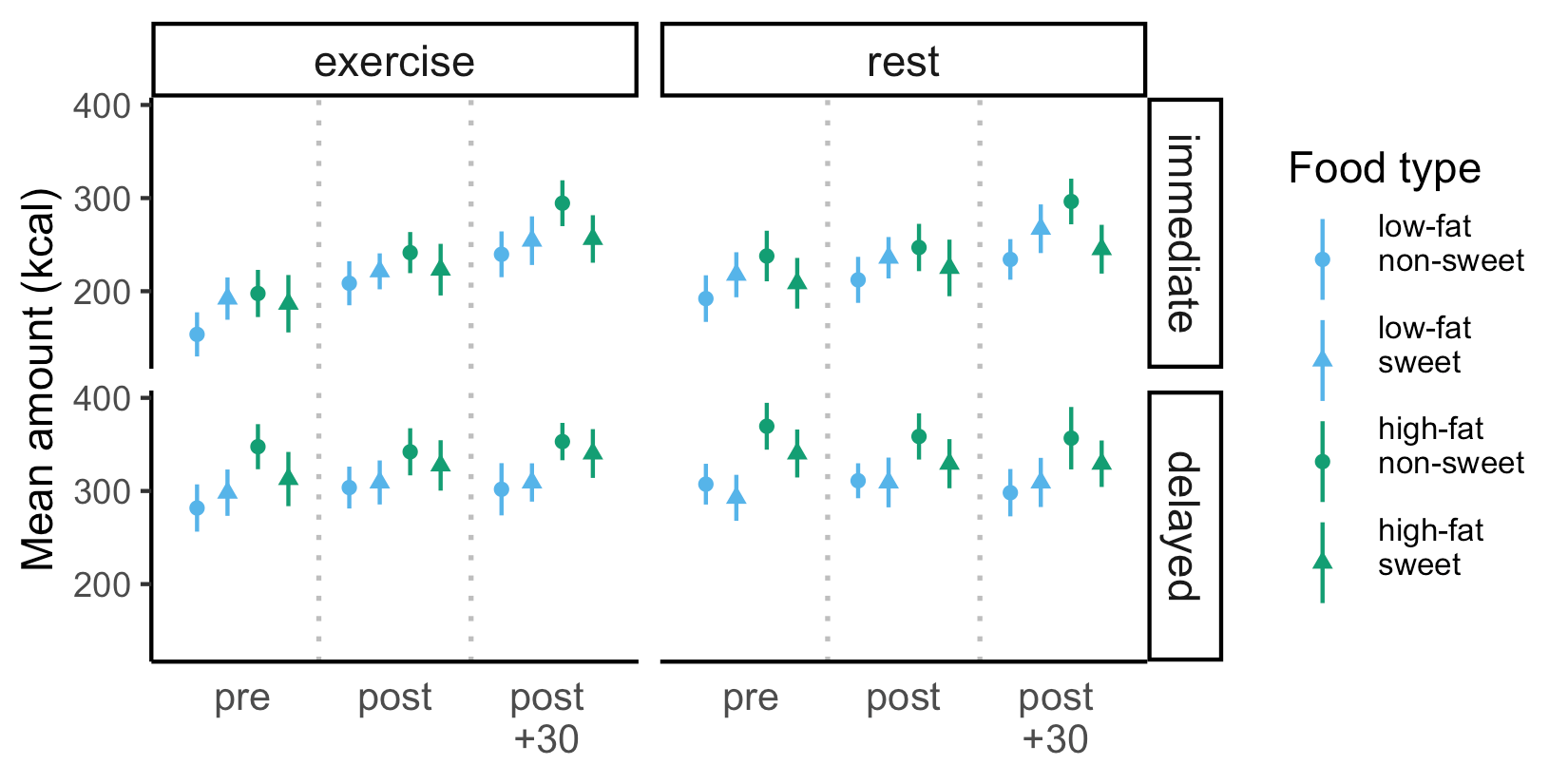

Supplement: Supplementary file 1 [file nutrients-13-00347-s001.zip › Figure S1.png]

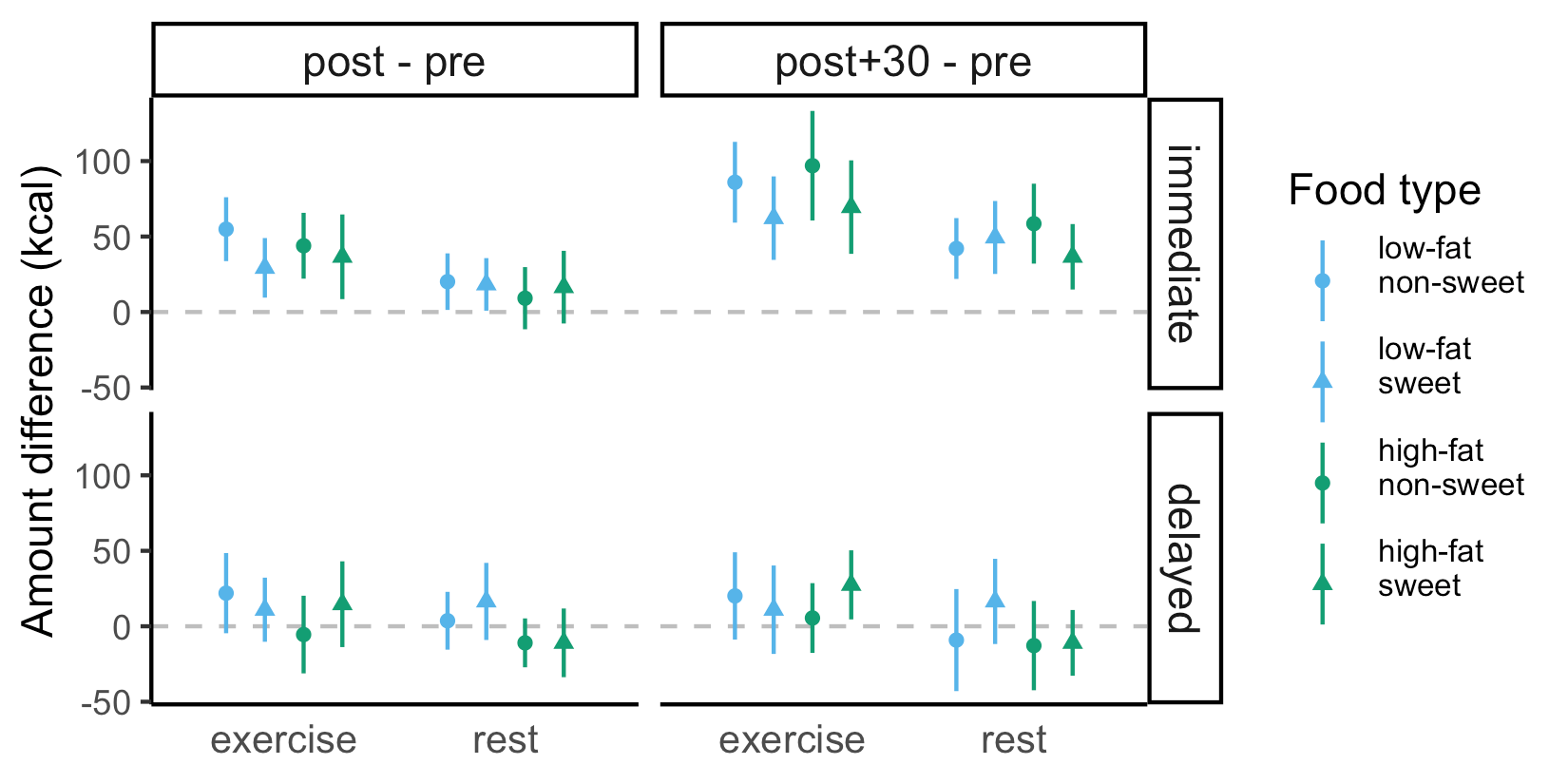

Supplement: Supplementary file 1 [file nutrients-13-00347-s001.zip › Figure S2.png]

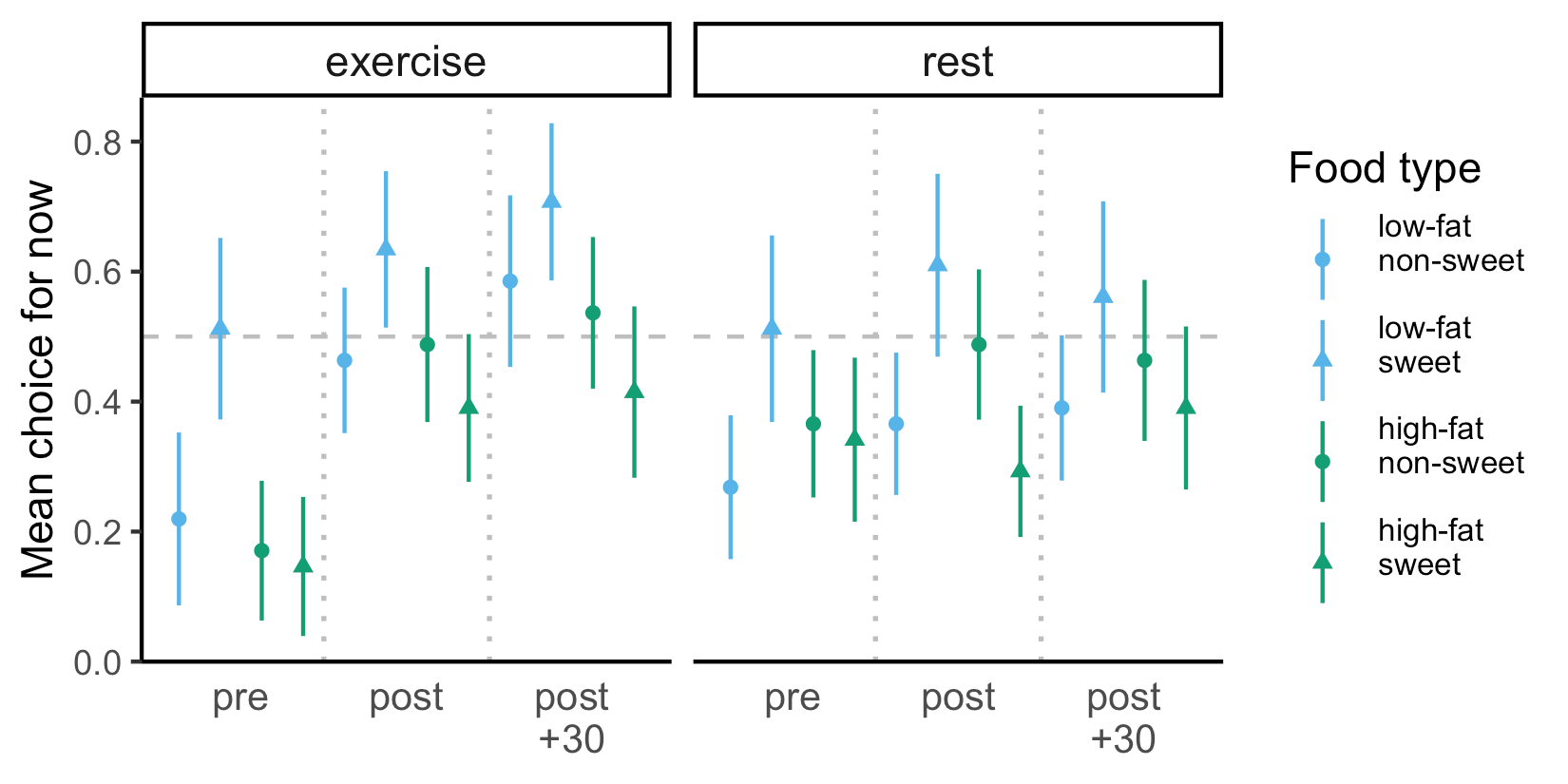

Supplement: Supplementary file 1 [file nutrients-13-00347-s001.zip › Figure S3.png]

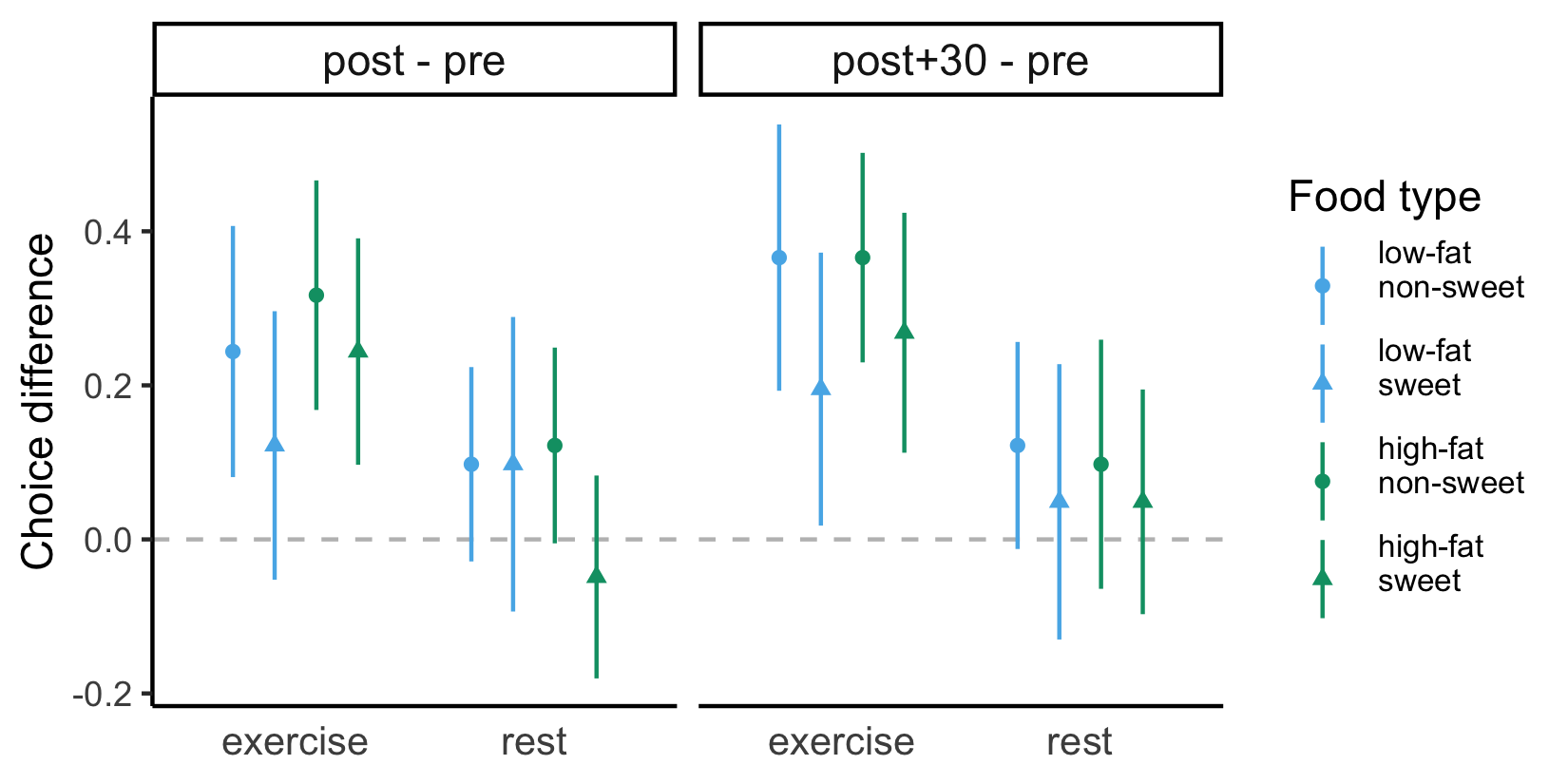

Supplement: Supplementary file 1 [file nutrients-13-00347-s001.zip › Figure S4.png]
