# Supplementary material for: Exercise Shifts Hypothetical Food Choices toward Greater Amounts and More Immediate Consumption
Source: Nutrients. 2021 Jan 24;13(2):347. doi: 10.3390/nu13020347 (PMC7911174; doi:10.3390/nu13020347)
Supplement: Supplementary file 1 [file nutrients-13-00347-s001.zip › Supplemental Digital Content 1.doc]

**CONSORT 2010 Flow Diagram**

**Allocation**

**Analysis**

**Follow-Up**

**Enrollment**

Assessed for eligibility (n = 69)

Excluded (n = 16)

- Failed to meet inclusion criteria (n = 11)
- Declined to participate (n = 1)
- Other reasons (n = 4)
  - Unfit to exercise (n= 3)
  - Declined to begin intervention (n = 1)

Lost to follow-up (n= 0)

Discontinued condition (n= 2)

- Illness (n= 1)
- Unexpected pregnancy (n= 1)

Allocated to condition (n= 53)

- Received allocated condition (n= 50)
- Did not receive allocated condition (n= 3)
  - Time constraints (n = 3)

Analysed (n= 41)

- Excluded from analysis (n= 7)
  - Age criteria updated (n= 1)
  - Exceeded exercise intensity (n= 6)

Randomized (n= 53)
